# Prediction the functional impacts of highly deleterious non-synonymous variants of *TSGA10* gene

**DOI:** 10.22099/mbrc.2024.49991.1977

**Published:** 2025

**Authors:** Zeinab Jamali, Mahsa Zargar, Mohammad Hossein Modarressi

**Affiliations:** Department of Medical Genetics, School of Medicine, Tehran University of Medical Sciences, Tehran, Iran

**Keywords:** Testis specific gene antigen 10, Non-synonymous mutation, Spermatogenesis, Cancer

## Abstract

Testis specific gene antigen 10 (TSGA10) is a protein which has roles in spermatogenesis and cancers so that deletion or mutation in the *TSGA10* gene resulted in non-obstructive infertility and aberrant expression of this protein, was detected in solid tumors and leukemia. Despite the crucial roles of TSGA10 in tumorigenesis and infertility, yet it is not obvious how various nsSNPs of its gene impress the structure and function of the TSGA10. Therefore, it is worthwhile to investigate the potential highly deleterious nsSNPs by several in-silico tools before launching costly experimental approaches. In the current study, we employed several different machine learning algorithms in a two-step screening procedure to analyze single nucleotide substitutions of *TSGA10* gene. Prediction tools were included SIFT, PROVEAN, PolyPhen-2, SNAP2, SNPs & GO, PhD-SNP for the first step and the second step included predictive tools such as I-mutant 3.0, MUpro, SNPeffect 4.0 (LIMBO, WALTZ, TANGO, FoldX), MutationTaster and CADD. Also, the 3D models of significantly damaging variants were built by Phyre2. The results elucidated 15 amino acid alterations as the most deleterious ones. Among these S563P, E578K, Q580P, R638L, R638C, R638G, R638S, L648R, R649C, R649H were located in a domain which is approved to has interaction with the HIF1-A protein and D62Y, R105G, D106V and D111Y were located on phosphodiesterase domain. In sum, these predicted mutations significantly influence the function of TSGA10 and they could be used for precise study of this protein in infertility and cancer experimental investigations.

## INTRODUCTION

Testis specific gene antigen 10 (TSGA10) is a protein with 698 amino acids and 82 kDa weight. Its gene is located on 2q11.2 and contains 19 exons Firstly, TSGA10 was described as a cancer testis antigen (CTA) in 2001 [1]. TSGA10 mRNA processed to produce two protein fragments, a 27 kDa N-terminal and a 55 kDa C-terminal fragments, which are located in the fibrous sheath of the sperm tail, and midpiece of sperm, respectively [2]. It is confirmed that TSGA10 has crucial roles in normal spermatogenesis and controlling malignancy. Low expression of TSGA10 in some high-grade tumors like nasopharyngeal carcinoma and esophageal squamous cell carcinomas was shown previously. 

**Figure 1:**
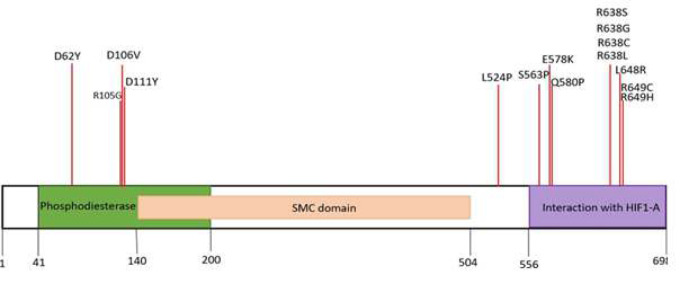
The positions of 15 significantly deleterious aa substitutions of TSGA10 relative to its domains.

Several domains are considered for TSGA10 protein. Amino acids 41-200, form a domain with putative phosphodiesterase activity; a C-terminal domain which interacts with HIF1A, consisted of residues 556 to 689 and a domain named “structural maintenance of chromosome protein 1” (SMC1) including 140-504 residues which have a centrosome related roles during anaphase and telophase (Fig. 1) [3, 4].

It is indicated that, overexpression of this protein can prevent the nuclear accumulation of hypoxia-inducible factor 1 (HIF-1α) specially using C-terminus fragment and trigger inhibitory effects on tumor angiogenesis and metastasis [2, 5]. HIF-1α localized into nucleus and then create a heterodimer formation with HIF-1β which lead to increase the expression of numerous target genes with different roles in metabolism, angiogenesis, apoptosis, iron metabolism, proliferation, invasion and metastasis [6, 7]. It is demonstrated that, TSGA10 interacts with the TAD-C and PAS-B domains of HIF-1 while the binding affinity to TAD-C is higher. Consequently, this interaction prevents binding p300/CBP to this domain and inhibit HIF-1α dimerization and localization in nucleus as well as metastasis [5]. 

There is an association between aberrant expression of TSGA10 and different conditions such as acute myeloid leukemia (AML) [8], acute lymphoblastic leukemia (ALL) [9], brain tumors, breast cancers, gastrointestinal tumors, skin tumors and soft tissue tumors [10]. Specially, TSGA10 expression is decreased in esophageal squamous cell carcinoma and breast cancer cells in comparison to normal controls which introduces it as a tumor suppressor gene [7]. It is indicated that *TSGA10* gene mutations such as c.211delG and c.545dupT are also associated with acephalic spermatozoa [11, 12]. 

According to the data we retrieved from dbSNP database at NCBI (http://www.ncbi.nlm. nih.gov/SNP/), *TSGA10* contains 34,675 different polymorphisms in different functional classes such as intronic variants, in frame deletions and insertions, initiator codon variants, missense, synonymous and non-coding transcript variants. Missense single nucleotide variants are 574. 

There are few investigations about the *TSGA10* polymorphisms and diseases. The association of rs200902126 (A>G), rs3811553 (C>T) and rs17852533 (C>A) SNPs and oligospermia and azoospermia were investigated previously in a case- control study and the results did not show any significant association in the studied population [13]. Although, it is hypothesized that the rs17852533 (C>A, Leucin to methionine ), may influence the proteolytic process of this protein and consequently alter its function [14]. non-coding variants also reported to effect on TSGA10 protein, for instance a splicing mutation (NM_025244: c.1108-1 G >T) which resulted to skipping the exon15 and defected the protein function and structure was reported in a family with acephalic spermatozoa [12].

So, there are yet many uncharacterized non synonymous variants related to this gene, which can be effective in male infertility and cancer. Because of the roles of this protein in crucial biological processes related to spermatogenesis and cancer, it is worthful to investigate the impacts of genomic variants of this gene on the function of corresponding protein. On the other hand, among a tremendous number of SNPs, only some of them can be damaging. So, the computational approaches could be useful to predict the impacts of a given variant before launching a time consuming and costly experimental study for investigation the effect of amino acid changes on the protein function.

## MATERIALS AND METHODS

**SNP data gathering: **nsSNPs of *TSGA10* gene were retrieve using dbSNP which is available through National Centre for biotechnology information (NCBI) (http://www.ncbi.nlm. nih.gov/). The Ensemble transcript ID ENST00000393483.8 and refseq match number NM_025244.4 was used for analysis the variants. The protein sequence also retrieved from UniProtKB database (http://www.uniprot.org/uniprot/) (Q9BZW7) for tools that need the protein sequence information. 

**Sorting Intolerant from Tolerant (SIFT): **SIFT algorithm which first introduced in 2001, predicts the impact of coding variants on the protein function. SIFT considers the protein conservation with homologous sequences and the severity of the amino acid alteration. The results are shown as “damaging” or “tolerated” according to the tolerated index [15]. It is available in http://sift.jcvi.org/. 

**Protein variation effect analyzer (PROVEAN): **PROVEAN is a suitable web server to predict the single or multiple amino acid changes or amino acid insertion/ deletions. Briefly, it uses supporting sequence set and generates a delta alignment score for each supporting sequence. The averages of scores within and across the clusters in sequence sets generate the PROVEAN score [16]. PROVEAN is available in http://provean.jcvi.org and the input type for PROVEAN prediction is a comma-separated residue-based system. 

**Polymorphism phenotyping V2 (PolyPhen-2): **PolyPhen-2, is available via webserver (http://genetics.bwh.harvard.edu/pph2/) or software. Using structural and comparative evolutionnary considerations, PolyPhen-2 analysis the potential impacts of amino acid alterations on the protein structure, function and its stability [17]. The variants classified qualitatively as “benign”, “possibly damaging” and “probably damaging”. 

**Screening for Non-Acceptable Polymorphisms (SNAP2):** Using evolutionary information for a given residue, prediction the secondary structure and annotations, SNAP2 predicts the functional changes due to the presence of a nsSNP based on a neutral network. The SNAP2 network needs protein sequences and lists of variants and gives a score for each nsSNP, which ultimately be translated into binary predictions of a “neutral” or “effect” [18].

**Prediction the protein stability changes using I-mutant 3.0: **I-mutant 3.0 is a protein stability status predictor which can be used for screening of disease-causing variants. Its prediction is based on the change in protein fold stability (ΔΔG) in the presence of a substitution. The input format can be protein amino-acid sequence or three-dimensional wild-type structure. In the present study the protein sequence was used as input and the stability prediction was perform in 25 ^o^C and pH 7. Reliability Index (RI) computed for each prediction and the results showed as “increase” or “decrease” terms. This tool is available in http://gpcr2. biocomp.unibo.it/cgi/predictors/I-Mutant3.0/I-Mutant3.0.cgi. 

**Protein stability prediction by MUpro: **Regarding the protein stability MUpro (http://mupro.proteomics.ics.uci.edu/) also predicted both the value of energy change (ΔΔG) and the sign of energy change using SVM and sequence information only. Accordingly, the results are shown as “increase” or “decrease”. 

**SNPeffect 4.0: **Crucial phenotypic properties of desired changes like aggregation propensity, stability changes, alterations in chaperon binding sites and amyloid formation can be achieved by using online predictor SNPeffect4.0 which is available at https://snpeffect. switchlab.org/. SNPeffect 4.0 is based on sequence- and structure of the protein and was used in the present study to effect of substitutions on the structural phenotype of TSGA10 protein. Three properties of a given protein in the presence of a substitution can be analyzed by this web server tool using three different algorithms. TANGO algorithm can predict some regions of the protein with the highest aggregation propensity according to the changes in hydrophobicity and prone beta-sheet forming regions, consequently, it reports the results of mutation effects as dTANGO scores, WALTZ predicts the amyloid propensity of the changed protein with the highest specificity and the results reported as dWALTZ, LIMBO can predict the chaperone binding sites for Hsp70 chaperones, and shows the if there is any changes in chaperon binging tendency and results are available as dLIMBO scores. At last, the structural modeling and protein stability analysis also performed by FoldX. The input format can be UniProt ID, FASTAsequence, or PDB file. In the present study the UniProt ID of TSGA10 protein (Q9BZW7) was used as input. 

**Mutation Taster: **Mutation Taster (http://www.mutationtaster.org/ChrPos.html) leverages multiple biomedical databases and established analysis tools. The results undergo evaluation by a Naive Bayes classifier, which effectively predicts whether a variant is disease-causing or a polymorphism. Mutation Taster integrates two different conservation predictive tools: phyloP and phastCons. phastCons values, ranging from 0 to 1, depict the likelihood that each nucleotide is part of a conserved element, while phyloP, with values between -14 and +6, individually measures conservation at specific columns, disregarding neighboring effects. Prediction of splice sites by Mutation Taster makes use of the NNSplice program [19].

**Analysis by the combined annotation dependent depletion (CADD): **CADD is available at https://cadd.gs.washington.edu/score. The CADD scores show the pathogenicity degree of a variant such as single nucleotide substitution and insertion/deletions in the human genome by combining several annotations such as conservation and functional data for a given change into one metric. So, CADD is considered as a comprehensive and reliable prediction tool. The input needed for prediction using CADD is a Variant Call Format (VCF) file including the chromosome position and the exact alteration [20].

**Prediction of three-dimensional structure of the wild-type and altered TSGA10 by** **Phyre2:** Phyre2 (http://www.sbg.bio.ic.ac.uk/phyre2/html/page.cgi?id=index) was used to predict the three-dimensional structure of the wild-type and mutant proteins, which is a Web Tool used to predict and investigate the function and structure of proteins or the 3D consequence of mutations. Its main aim is to offer researchers an easy-to-use interface for cutting-edge protein bioinformatics servers [21]. Phyre2 utilizes advanced methods based on the detection of homology to construct 3D models, forecast ligand binding sites, and assess the impact of amino acid variants of a given protein sequence [21]. The TSGA10 protein sequence was used as query and the modelling mode was chosen “normal”. 

## Results

There are 34675 SNPs for the *TSGA10* gene in NCBI dbSNP database including 33591 Intronic variants. Out of all SNPs, 574 were classified as non-synonymous (ns) SNPs and 226 SNPs as synonymous. Also, there are some tri- and four- allelic SNPs, by accounting them the overall number of nsSNPs get raised to 697. Some of these variants are positioning in intronic region for *TSGA10* transcript variant 1, so they do not have any impact on protein function or structure. Eliminating these no-impact variants resulted in ultimately 481 nsSNPs for in-silico analysis. The ensemble *TSGA10* transcript ID used in this study was ENST00000393483.8. The RefSeq transcript number was NM_025244.4 and the RefSeq protein number was NP_079520.1. The UniProt ID for the corresponding protein (isoform1) was Q9BZW7.

The SIFT algorithm is based on the homologues protein sequence alignment so this tool can predict the effect of amino acid substitutions on the protein function. The probability scores less than 0.05 are determined as damaging. The results of all 481 submitted nsSNP rsIDs in SIFT showed that 43 (8.93%) variants are among the damaging predicted nsSNPs with scores between 0-0.028. The remaining 438 (91.06%) nsSNPs are predicted as tolerated variants with scores 0.05 or > 0.05. Some of the obtained results of SIFT and the related scores are shown in Table1. 

Another prediction tool based on alignment scoring tool is PROVEAN. The cutoff for prediction is -2.5. So, the variants with scores above -2.5 is considered as neutral and below or equal scores predicted as deleterious. The results of PROVEAN showed that from 481 variants, 41 nsSNPs are deleterious for protein biological function. The scores are between -2.56 and -7.14. Among these deleterious variants 38 nsSNPs overlapped with SIFT damaging results. The remaining 440 variants are predicted as “neutral” by PROVEAN because of their >-2.5 scores. Table1 contains some of the PROVEAN predictions and their scores. 

Polyphen-2 can predict the impact of the amino acid substitution on a given protein structure or function using straightforward evolutionally and comparative approach. There are three probability classification for the results of Polyphen-2 according to the PSIC score. The scores >0.85 are predicted as “probably damaging”, variants with the scores >0.15 are considered as “possibly damaging” and the others predicted as “benign” variants. The results of this tool demonstrated that out of 481 submitted variants 179 and 96 variants are predicted as “probably damaging” and “possibly damaging” respectively and the remaining 206 variants are “benign” predicted ones. 117 damaging or deleterious variants are shared between SIFT and PROVEN. Among these,103 nsSNPs are also classified as probably or possibly damaging by PolyPhen-2. So, obviously, there is a significant correlation between the two homology based tools (SIFT and PROVEAN) and the structural and physical based tool (PolyPhen-2). Table 1 indicates some of the results of polyphen-2 for nsSNPs. 

**Figure 2:**
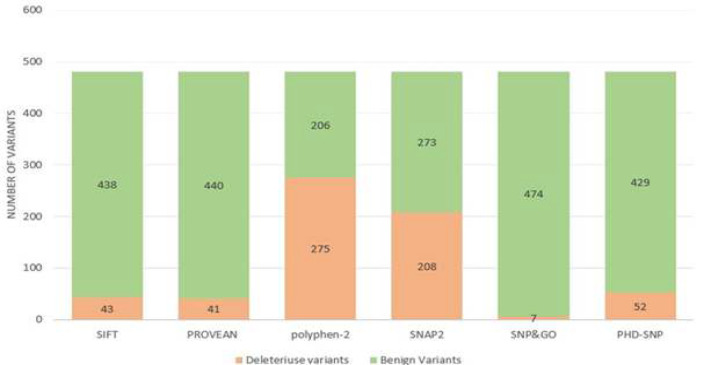
The results of the prediction of six first step refinement tools

Predicting the disease associated variants was performed using SNPs&GO. Using the protein sequence or the Swiss-Prot code of the protein (Q9BZW7), this tool screened the amino-acid substitutions and the output is shown according to the SNPs&GO and PhD-SNP as “Disease” or “Neutral” mutations. From 481 variants, 7 and 52 substitutions are predicted as “disease” by SNPs&GO and PhD-SNP respectively. SNAP2 online tool predicted that from 481 variants, 266 aminoacid changes have “neutral” impact on the protein and 208 alterations predicted as “effect” ones. five remaining alterations include C21S,M82I,M329I,N438K,Q142H were considered as repetitive aminoacid changes because of codon degeneracy. Also, there were two stop codons without any impacts on the aminoacid alteration (Q433*,R679*). Figure 2 showed the results of all six prediction tools based to the number of deleterious or benign variants.

To achieve the results of the first refinement step, the out puts of the all six evolutionary and SVM based predicting tools combined to each other. So, after combining the results of SIFT, PROVEAN, Polyohen-2, PhD-SNP, SNP&GO and SNAP2, variants which are predicted to have a deleterious functional effect on the protein by at least five tools, were selected. Accordingly, 40 mutations were classified as deleterious, damaging or disease-causing variants. Additionally, R638L, R650P, R649C, R638G, R638S were predicted as damaging mutations according to the results of all six servers (Table 1). All of these 40 nsSNPs were selected for further analysis. 

I-Mutant Suite provide the opportunity to predict the stability changes of a given protein in the presence of single-site mutations. The results of analysis of the TSGA10 protein in the pH 7.0 and temperature of 25^o^C in the presence of each 40 variants showed that four amino acid substitutions (S510P, S563P, D111Y, K270N) increased and the remaining 36 substitutions decreased the stability of protein respectively. The results are shown in the supplementary Table 1.

For each potential mutation the value and sign of energy change predicted using SVM and sequence information. According to the results two mutations increased the stability of TSGA10 protein considering delta delta G (R638L and R330L) and remaining 38 ones decreased the protein stability (Supplementary Table 1). 

 The online predictor SNPeffect 4.0 was used to achieve the crucial phenotypic properties of desired amino acid substitution like stability changes, aggregation propensity and changes in chaperon binding sites. The results are shown in supplementary table 1 for each of 40 amino acid substitutions. According to the results of LIMBO, none of amino acid changes had any impact on Chaperone binding tendency of TSGA10 protein. The results of WALTZ predictor indicated that D111Y and R105G increase the amyloid propensity of TSGA10 with dWALTZ scores 429.25 and 260.57 respectively and D62Y decrease this feature with the score -76.79. TANGO results also showed that the aggregation tendency of protein increased in the presence of D111Y and D106V with the 203.50 and 182.93 dTANGO scores respectively. FoldX results do not show any changes in TSGA10 stability. The supplementary table 1 shows the results for every four predictors. 

MutationTaster analyzes the influence of an amino acid substitution on the protein evolutionary conservation, splice-site changes, loss of protein features and changes that might affect the amount of mRNA. This tool indicated that 11/40 amino acid alterations are deleterious. The conservation analysis of all 40 variants shows that the PhastCons scores for 38 substitutions are 1 and the scores for R331W and R330L were 0.669 and 0.996 respectively. The Phylop scores for substitutions are between 0.047 and 4.494, while the scores of 19 of them are above 3.0. So, all of the Variants are belonged to a conserved element of genome according to the PhastCons (1 or near 1 results) and Phylop (because of positive scores of variants) results.

PHRED-scaled scores in CADD (C-score) helps the interpretation of the deleteriousness of a variant. C-scores equal or greater than 10 means, these substitutions are predicted among the 10% most deleterious variants in human genome, a C-score 20 or greater shows the 1% most deleterious substitutions and when it is 30 or greater than 30 means the variants are in the top 0.1% deleterious ones. In the present study all of the 40 refined variants have a CADD score above 20 and the scores for E491K, D278G, R330C, R649C and R638C mutations were above 30 which indicates that the variants are predicted as the 0.1% most deleterious in genome. 

**Table 1:** Describing the results of first refinement step of nsSNP analysis

	**Aa change**	**SIFT prediction**	**SIFT scores**	**PROVEAN results**	**PROVEAN** **Scores **	**PolyPhen-2 results**	**SNAP prediction**	**SNPs&GO**	**PhD-SNP**
1	E44K	Damaging	0.002	Deleterious	-3.43	PD	Effect	Neutral	Disease
2	R158P	Damaging	0.003	Deleterious	-4.32	PD	Effect	Neutral	Disease
3	S510P	Damaging	0.003	Deleterious	-3.7	PD	Effect	Neutral	Disease
4	R617G	Damaging	0.001	Deleterious	-5.31	PD	Effect	neutral	Disease
5	L112S	Damaging	0.001	Deleterious	-4.41	PD	Effect	neutral	Disease
6	S563P	Damaging	0.01	Deleterious	-2.56	PD	Effect	neutral	Disease
7	E339K	Damaging	0.003	Deleterious	-3.33	PD	Effect	neutral	Disease
8	D111Y	Damaging	0.001	Deleterious	-6.91	PD	Effect	neutral	Disease
9	L524P	Damaging	0.022	Deleterious	-3.19	PD	Effect	neutral	Disease
10	R331W	Damaging	0.00	Deleterious	-6.21	PD	Effect	neutral	Disease
11	E104A	Damaging	0.003	Deleterious	-4.75	PD	Effect	neutral	Disease
12	D106V	Damaging	0.001	Deleterious	-5.88	PD	Effect	Neutral	Disease
13	D106G	Damaging	0.005	Deleterious	-4.69	PD	Effect	neutral	Disease
14	V459G	Damaging	0	Deleterious	-5.09	PD	Effect	neutral	Disease
15	E491K	Damaging	0	Deleterious	-3.47	PD	Effect	neutral	Disease
16	E118G	Damaging	0.002	Deleterious	-5.98	PD	Effect	neutral	Disease
17	D62Y	Damaging	0.001	Deleterious	-7.14	PD	Effect	neutral	Disease
18	D278G	Damaging	0.002	Deleterious	-5.75	PD	Effect	neutral	Disease
19	D612Y	Damaging	0.001	Deleterious	-4.43	PD	Effect	neutral	Disease
20	E578K	Damaging	0.003	Deleterious	-3.13	PD	Effect	neutral	Disease
21	R45L	Damaging	0.013	Deleterious	-3.81	PD	Effect	neutral	Disease
22	K270N	Damaging	0	Deleterious	-4	PD	Effect	neutral	Disease
23	R330C	Damaging	0	Deleterious	-6.07	PD	Effect	Neutral	Disease
24	R638L	Damaging	0.002	Deleterious	-5.51	PD	Effect	Disease	Disease
25	R638H	Damaging	0.002	Deleterious	-3.95	PD	Effect	Neutral	Disease
26	R650P	Damaging	0.005	Deleterious	-5.05	PD	Effect	Disease	Disease
27	R650H	Damaging	0.003	Deleterious	-3.56	PD	Effect	Neutral	Disease
28	L648R	Damaging	0.001	Deleterious	-3.24	PD	Effect	Neutral	Disease
29	K55Q	Damaging	0.005	Deleterious	-3.21	PD	Effect	Neutral	Disease
30	R649C	Damaging	0	Deleterious	-3.26	PD	Effect	Disease	Disease
31	R638C	Damaging	0	Deleterious	-6.3	PD	Effect	Neutral	Disease
32	R638G	Damaging	0.001	Deleterious	-5.48	PD	Effect	Disease	Disease
33	R638S	Damaging	0.001	Deleterious	-4.45	PD	Effect	Disease	Disease
34	Q580P	Damaging	0.004	Deleterious	-4.12	PD	Effect	Neutral	Disease
35	R100P	Damaging	0.129	Deleterious	-3.68	PD	Effect	Disease	Disease
36	N566K	Damaging	0.003	Deleterious	-4.52	PD	Effect	Neutral	Disease
37	Q479H	Damaging	0.001	Deleterious	-4.5	PD	Effect	Neutral	Disease
38	R649H	Damaging	0	Deleterious	-3.89	PD	Effect	neutral	Disease
39	R105G	Tolerated	0.058	Deleterious	-4.24	PD	Effect	Disease	Disease
40	R330L	Damaging	0.003	Deleterious	-5.26	PD	Effect	Neutral	Disease

Note: PD means Probably damaging

The second refinement tools were consisting of I-mutant 3.0, MUpro, TANGO, WALTZ, LIMBO, FoldX, MutationTaster and CADD. The conservancy of protein in the presence of a given variant was also predicted using phastCons and phyloP. According to the results of predictions, 15 substitutions were predicted to alter the protein structure. The deleteriousness effect of these 15 variants were confirmed in at least three above mentioned prediction tools. These aminoacide alterations were D62Y, R105G, D106V, D111Y, L524P, S563P, E578K, Q580P, R638L, R638C, R638G, R638S, L648R, R649C, and R649H (Table 2).

The wild-type and mutant proteins' three-dimensional structures were predicted using Phyre 2, and the results are depicted in supplementary figure. The confidence and coverage for predicting the native TSGA10's three-dimensional structure were 99.7% and 93%, respectively. For the 15 predicted mutant TSGA10 proteins, the confidence scores ranged between 95% and 99.6%, and the coverage ranged between 63% and 99% (refer to Supplementary Table 2).

**Table 2:** The results of second refinement step for identification of the most significant substitutions (only 15 deleterious variants are shown).

							**SNP effect**			**Conservancy**	
**AA** **change**		**CADD**	**Mutation** **Taster**	**I-mutant-3**	**MUpro**	**FoldX Protein** **stability**	**LIMBO Chaperone binding** **tendency (dLIMBO score**		**WALTZ Amyloid propensity** **(dWALTZ score)**	**TANGO Aggregation tendency** **(dTANGO score**	**Phylop**	**PhastCons**
**D62Y**		24.7	Benign	Decrease	DECREASE	No structural information	not affected (0.00)		Decreased (-76.79)	Not affected (32.39)	1.918	1
**R105G**		25.9	Benign	Decrease	DECREASE	No structural information	not affected (0.00)		Increased (260.57)	Not affected (-0.28)	1.592	1
**D106V**		26.8	Benign	Decrease	DECREASE	No structural information	not affected (0.00)		Not affected (-7.51)	Increase (182.93)	3.487	1
**D111Y**		28.6	Benign	Increase	DECREASE	No structural information	not affected (0.00)		Increase (429.25)	Increase (203.50)	4.11	1
**L524P**		23.3	Deleterious	Decrease	DECREASE	No structural information	not affected (0.00)		not affected (0.00)	Not affected (0.00)	3.607	1
**S563P**		25.1	Deleterious	Increase	DECREASE	No structural information	not affected (0.00)		Not affected (-0.01)	Not affected (1.21)	1.421	1
**E578K**		29.9	Deleterious	Decrease	DECREASE	No structural information	not affected (0.00)		Not affected (-0.14)	Not affected (0.23)	4.494	1
**Q580P**		26.3	Deleterious	Decrease	DECREASE	No structural information	not affected (0.00)		Not affected (-1.24)	Not affected (-0.40)	3.731	1
**R638L**		29.2	Deleterious	Decrease	INCREASE	No structural information	not affected (0.00)		Not affected (2.88)	Not affected (-0.28)	4.102	1
**R638C**		31	Deleterious	Decrease	DECREASE	No structural information	not affected (0.00)		Not affected (0.14)	Not affected (-0.28)	2.442	1
**R638S**		27.2	Deleterious	Decrease	DECREASE	No structural information	not affected (0.00)		Not affected (0.15)	Not affected (-0.28)	2.442	1
**R638G**		28.7	Deleterious	Decrease	DECREASE	No structural information	not affected (0.00)		Not affected (0.14)	Not affected (-0.28)	2.442	1
**L648R**		27.2	Deleterious	Decrease	DECREASE	No structural information	not affected (0.00)		Not affected (-0.14)	Not affected (0.28)	3.559	1
**R649C**		32	Deleterious	Decrease	DECREASE	No structural information	not affected (0.00)		Not affected (0.01)	Not affected (-0.28)	4.292	1
**R649H**		26.7	Deleterious	Decrease	DECREASE	No structural information	not affected (0.00)		Not affected (2.06)	Not affected (-0.24)	2.782	1

## DISCUSSION

In the present study 481 missense single nucleotide variants out of 34675 SNPs of the *TSGA10* gene were analyzed using several different bioinformatic web-based tools. The analysis was performed in a two-step refinement procedure. The results of the first step provide 40 amino acid changes with the highest influence on TSGA10 protein function and the second step results were consisted of 15 amino acid variants with the most significant deleteriousness effect on the biological functions or structure of the protein. 

TSGA10 protein was consider as a kind of cancer testis antigens previously. But it was shown that TSGA10 has a broadly expression in normal tissues, too. So, it does not seem correct to consider it as a CTA [5]. However, the role of this protein in spermatogenesis is demonstrated previously so that deletion or mutation in the *TSGA10* gene can lead to non-obstructive infertility. Additionally, aberrant expression of TSGA10, was detected in solid tumors and leukemia in several studies[9, 22]. Amoorahim et al. showed that TSGA10 expression inhibit endothelial cells to proliferate and migrate and it can suppress angiogenesis [23]. So, the expression of TSGA10 is a common feature of both cancer cells and testis tissue cells which have high levels of proliferation in the hypoxia condition. Hypoxia inducible factors (HIFs) are overexpressed in the response to the hypoxia condition. Because of normal function of testicular cells without any cancerous status, it is probable that factors such as TSGA10 protein inhibit the HIF function and consequently cancer development [23]. It is demonstrated that, TSGA10 inactivates the HIF-1α by docking to the PASB and TAD-C domains of this protein which lead to inhibition the binding of P-300 co-activator and ultimately prevent HIF-1 dimerization and its activation [5]. 

Amino acid changes which are due to single nucleotide variants such as nonsynonymous single nucleotide polymorphisms (nsSNP), can be deleterious for protein structure and function or even be disease-causing [24]. Because of the roles of TSGA10 in spermatogenesis and cancers any changes in this protein can be related to different kinds of conditions. Although, it is a time consuming, costly and effortful to screen all nsSNPs in a gene, experimentally. Rationally, not all of nsSNPs have deleterious effect, so it is better to first investigate the potential damaging variants using recently developed computational predictor tools [25]. Therefore, in the present study all of coding non synonymous SNPs of *TSGA10* analyzed using multiple structure and sequence based powerful online predictor tools in a two-step refinement procedure. The first step consisted of SIFT, PROVEAN, PolyPhen-2, SNAP2, SNPs & GO, PhD-SNP and the results of this step indicated that from all 481 missense variants, 40 variants potentially had a damaging effect on protein by prediction of at least five above mention tools (Table1). The second step included predictive tools such as I-mutant 3.0, MUpro, SNPeffect 4.0 (LIMBO, WALTZ, TANGO, FoldX), MutationTaster and analysis by CADD. After this refinement procedure we reached to the 15 amino acid changes which can influence the stability of TSGA10 protein, the aggregation tendency, changed the chaperon binding regions, changed the amyloid formation propensity, and ultimately, considered as disease causing substitutions. Also, the 3D models of these 15 variants were built by Phyr2. 

According to the SIFT results the 14/15 variants were damaging and the scores were between 0-0.022. R105G (SIFT score: 0.058) was predicted as tolerated variant in SIFT. All of variants are predicted to be deleterious by PROVEAN (Scores were between -7.14 for D62Y and -2.56 for S563P) and 14/15 substitutions were considered as “Probably damaging” while L524P was predicted as “Possibly damaging”. I-mutant 3.0 and MUpro servers were used to assessing the stability of TSGA10 protein in the presence of amino acid substitutions. Amino acid alterations, which change the hydrophobicity, folding, backbone strain, or alter the interactions, influence stability and biological functions of a protein, while other substitutions can be tolerated [26]. According to I-mutant prediction, S510P, S563P, D111Y and K270N increased and other amino acid changes decreased the stability while MUpro predicted two others (R638L, R330L) as stability enhancing variants and the other 13 ones as stability reducing. Hence the stability of TSGA10 get raised in the presence of six new amino acids Tyrosine, Asparagine, Leucine, Proline, Proline and Leucine in the positions 111, 270, 330, 510, 563 and 638 respectively. Mansouri et al. showed that when its levels get to a significant threshold, TSGA10 protein have antiangiogenic and anti-metastatic functions through HIF-1[5]. Therefore, any changes in its amino acid composition which enhances TSGA10 stability also can help to its anti-tumoral effects. Although experimental analysis needs to confirm this. From 15 highly damaging amino acid changes D106V, D111Y, D62Y and R105G are located on Phosphodiesterase domain (Fig. 1). In all three first alterations aspartic acid is replaced with Valine and Tyrosine, so an acidic hydrophile amino acid is replaced by two hydrophobic ones in 62,106 and 111 positions. There are several previously reports indicated that mutations in the Phosphodiesterase domain can lead to acephalic spermatozoa including frame shift mutation c.545dupT:p.Ala183Serfs* reported in a patient with heterozygous parents(10). Meanwhile, according to the conservancy predicted tools, Phylop and PhastCons D106, D111, D62 are conserved positions. Also, according to the results, D106V, D111Y increased the aggregation tendency while D111Y increased the Amyloid propensity of the TSGA10 so it probable that these two substitutions be involve in pathogenesis related to TSGA10 specially in acephalic spermatozoa. 

 According to the UniProtKB database and previous studies amino acids 556-689 (TSGA10 C-terminal) interact with HIF1-A [28]. 10/15 significantly deleterious amino acid alterations including S563P, Q580P, E578K, R638L, R638C, R638G, R638S, L648R, R649C, R649H were located in this domain and are responsible for interaction of TSGA10 with HIF1-A. from these, R638 and R649 are relatively hot spot altered positions, so that Arginine in position 638 is replaced by leucine, cysteine, glycine and serine and Arginine 649 is substituted with cysteine and histidine. Substitution of R638 with each of four amino acids cause reduction in protein stability, although these variants do not affect the amyloid propensity or aggregation tendency. When a positive charged arginine residue is replaced by a polar uncharged one like serin or un polar residues like leucine, cysteine, glycine, the hydrophobicity, hydrogen binds and the structure of protein may be affected so, it influenced the interactions between amino acids and then decrease the stability of protein. Although R649 is a conserved position and its substitu-tions decreased the stability of TSGA10 but it is less probable that its alteration impress the function of protein specially about R649H because of the similarly between Arginine and histidine residues. Although replacement to cysteine can influence the disulfide bonds in the protein or TSGA10 and HIF1-A interaction. Replacement of serin with a more hydrophobic residue like proline can change the protein function because of production a more stable protein. So, I mutant 3.0 predicted that S563P enhances the protein stability (RI = 5) although, the 3D structure of this variant is very different from wild-type protein (supplementary figure). The other alteration Q580P (glutamine to proline), decreased the stability because Proline can disrupt the secondary structure of TSGA10 [28]. In E578K a negatively charged residue, glutamic acid is replaced by a positive one, lysine. Although both glutamic acid and lysin residues have hydrophilic properties but lysine has hydrogen donor atoms in its side chain and glutamic acid is a hydrogen bond acceptor. Furthermore, glutamic acid play roles in forming ionic bonds or salt bridges in the protein. So, this alteration can significantly disrupt the packaging function of TSGA10. 

## References

[1] Modarressi MH, Cameron J, Taylor KE, Wolfe J (2001). Identification and characterisation of a novel gene, TSGA10, expressed in testis. Gene.

[2] Kazerani R, Salehipour P, Shah Mohammadi M, Amanzadeh Jajin E, Modarressi MH (2023). Identification of TSGA10 and GGNBP2 splicing variants in 5' untranslated region with distinct expression profiles in brain tumor samples. Front Oncol.

[3] Behnam B, Mobahat M, Fazilaty H, Wolfe J, Omran H (2015). TSGA10 is a centrosomal protein, interacts with ODF2 and localizes to basal body. J Cell Sci Therapy.

[4] Xiang M, Wang Y, Xu W, Zheng N, Zhang J, Duan Z, Zha X, Shi X, Wang F, Cao Y, Zhu F (2021). Pathogenesis of acephalic spermatozoa syndrome caused by splicing mutation and de novo deletion in TSGA10. J Assist Reprod Genet.

[5] Mansouri K, Mostafie A, Rezazadeh D, Shahlaei M, Modarressi MH (2016). New function of TSGA10 gene in angiogenesis and tumor metastasis: a response to a challengeable paradox. Hum Mol Genet.

[6] Jamali Z, Taheri-Anganeh M, Entezam M (2020). Prediction of potential deleterious nonsynony-mous single nucleotide polymorphisms of HIF1A gene: A computational approach. Comput Biol Chem.

[7] Jahani M, Shahlaei M, Norooznezhad F, Miraghaee SS, Hosseinzadeh L, Moasefi N, Khodarahmi R, Farokhi A, Mahnam A, Mansouri K (2020). TSGA10 over expression decreases Metastasic and metabolic activity by inhibiting HIF-1 in breast Cancer cells. Arch Med Res.

[8] Hoseinkhani Z, Rastegari-Pouyani M, Oubari F, Mozafari H, Rahimzadeh AB, Maleki A, Amini S, Mansouri K (2019). Contribution and prognostic value of TSGA10 gene expression in patients with acute myeloid leukemia (AML). Pathol Res Pract.

[9] Mobasheri MB, Modarressi MH, Shabani M, Asgarian H, Sharifian RA, Vossough P, Shokri F (2006). Expression of the testis-specific gene, TSGA10, in Iranian patients with acute lymphoblastic leukemia (ALL). Leuk Res.

[10] Mobasheri MB, Jahanzad I, Mohagheghi MA, Aarabi M, Farzan S, Modarressi MH (2007). Expression of two testis-specific genes, TSGA10 and SYCP3, in different cancers regarding to their pathological features. Cancer Detect Prev.

[11] Sha YW, Sha YK, Ji ZY, Mei LB, Ding L, Zhang Q, Qiu PP, Lin SB, Wang X, Li P, Xu X, Li L (2018). TSGA10 is a novel candidate gene associated with acephalic spermatozoa. Clin Genet.

[12] Ye Y, Wei X, Sha Y, Li N, Yan X, Cheng L, Qiao D, Zhou W, Wu R, Liu Q, Li Y (2020). Loss-of-function mutation in TSGA10 causes acephalic spermatozoa phenotype in human. Mol Genet Genomic Med.

[13] Soltani N, Yazarlou F, Akhondi MM, Sobhani M, Modarressi MH, Ghafouri-Fard S (2019). Certain TSGA10 polymorphisms are not associated with male infertility in Iranian population. Gene Rep.

[14] Modarressi MH, Ranjzad F, Tavallaei M, Asadi A, Zaim-Kohan H, Masoudi-Nejad A (2008). Importance of 273rd residue in proteolytic processing for production of functional TSGA10 protein. Biosci Hypotheses.

[15] Vaser R, Adusumalli S, Leng SN, Sikic M, Ng PC (2016). SIFT missense predictions for genomes. Nat Protoc.

[16] Choi Y, Chan AP (2015). PROVEAN web server: a tool to predict the functional effect of amino acid substitutions and indels. Bioinformatics.

[17] Adzhubei I, Jordan DM, Sunyaev SR (2013). Predicting functional effect of human missense mutations using PolyPhen‐2. Curr Protoc Hum Genet.

[18] Hepp D, Gonçalves GL, De Freitas TRO (2015). Prediction of the damage-associated non-synonymous single nucleotide polymorphisms in the human MC1R gene. PLoS One.

[19] Schwarz JM, Cooper DN, Schuelke M, Seelow D (2014). MutationTaster2: mutation prediction for the deep-sequencing age. Nat Methods.

[20] Rentzsch P, Witten D, Cooper GM, Shendure J, Kircher M (2018). CADD: predicting the deleteriousness of variants throughout the human genome. Nucleic Acids Res.

[21] Kelley LA, Mezulis S, Yates CM, Wass MN, Sternberg MJ (2015). The Phyre2 web portal for protein modeling, prediction and analysis. Nat Protoc.

[22] Behnam B, Chahlavi A, Pattisapu J, Wolfe J (2009). TSGA10 is specifically expressed in astrocyte and over-expressed in brain tumors. Avicenna J Med Biotechnol.

[23] Amoorahim M, Valipour E, Hoseinkhani Z, Mahnam A, Rezazadeh D, Ansari M, Shahlaei M, Hosseiny Gamizgy Y, Moradi S, Mansouri K (2020). TSGA10 overexpression inhibits angiogenesis of HUVECs: A HIF-2α biased perspective. Microvasc Res.

[24] Wu J, Jiang R (2013). Prediction of deleterious nonsynonymous single-nucleotide polymorphism for human diseases. ScientificWorldJournal.

[25] Badgujar NV, Tarapara BV, Shah FD (2019). Computational analysis of high-risk SNPs in human CHK2 gene responsible for hereditary breast cancer: A functional and structural impact. PLos One.

[26] Yang Y, Chen B, Tan G, Vihinen M, Shen B (2013). Structure-based prediction of the effects of a missense variant on protein stability. Amino Acids.

[27] Asgharzadeh MR, Pourseif MM, Barar J, Eskandani M, Jafari Niya M, Mashayekhi MR, Omidi Y (2019). Functional expression and impact of testis-specific gene antigen 10 in breast cancer: a combined in vitro and in silico analysis. Bioimpacts.

[28] Morgan AA, Rubenstein E (2013). Proline: the distribution, frequency, positioning, and common functional roles of proline and polyproline sequences in the human proteome. PLoS One.

