# Supplementary material for: Prediction the functional impacts of highly deleterious non-synonymous variants of TSGA10 gene
Source: Mol Biol Res Commun. 2025;14(1):47–58. doi: 10.22099/mbrc.2024.49991.1977 (PMC11624612; doi:10.22099/mbrc.2024.49991.1977)
Supplement: Supplementary file 1 — Table S1 [file mbrc-14-47-s001.pdf]

Table S1: The results of second refinement step for identification of the most significant substitutions (for all 40 variants)

	**Aa change**	**CADD**	**Mutation Taster**	**I-mutant-3**	**RI**	**MUpro**	**Delta G**	**SNP effect**				**Conservancy**	
								**FoldX Protein stability**	**LIMBO Chaperone binding tendency (dLIMBO score)**	**WALTZ Amyloid propensity (dWALTZ score)**	**TANGO Aggregation tendency (dTANGO score**	**Phylop**	**PhastCons**
1	E44K	23	Benign	Decrease	7	Decrease	-0.8585552	No structural information	not affected (0.00)	not affected (-0.87)	not affected (0.28)	2.916	1
2	R158P	28	Benign	Decrease	4	DECREASE	-0.88315353	No structural information	not affected (0.00)	Not affected (0.14)	(Not affected) -0.28	2.222	1
3	S510P	26.6.	Benign	Increase	3	DECREASE	-0.98778734	No structural information	not affected (0.00)	not affected (0.00)	Not affected (0.01)	3.607	1
4	R617G	22.9	Benign	Decrease	7	DECREASE	-2.2641057	No structural information	not affected (0.00)	Not affected (0.14)	Not affected (-4.91)	0.225	1
5	L112S	26.1	Benign	Decrease	9	DECREASE	-1.5841774	No structural information	not affected (0.00)	Not affected (0.11)	not affected (0.00)	3.41	1
6	**S563P**	**25.1**	**Deleterious**	**Increase**	**5**	**DECREASE**	**-1.2882062**	**No structural information**	**not affected (0.00)**	**Not affected (-0.01)**	**Not affected (1.21)**	**1.421**	**1**
7	E339K	28.7	Benign	Decrease	7	DECREASE	-1.0780254	No structural information	not affected (0.00)	Not affected (-0.12)	Not affected (4.09)	3.621	1
8	**D111Y**	**28.6**	**Benign**	**Increase**	**2**	**DECREASE**	**-0.098220634**	**No structural information**	**not affected (0.00)**	**Increase (429.25)**	**Increase (203.50)**	**4.11**	**1**
9	**L524P**	**23.3**	**Deleterious**	**Decrease**	**5**	**DECREASE**	**-1.9531981**	**No structural information**	**not affected (0.00)**	**not affected (0.00)**	**not affected (0.00)**	**3.607**	**1**

Table S1: The results of second refinement step for identification of the most significant substitutions (for all 40 variants)

10	R331 W	26.4	Benign	Decreas e	5	DECREA SE	-1.2872594	No structural informati on	not affected (0.00)	Not affected (0.39)	Not affected (- 0.25)	0.047	0.669
11	E104 A	26.3	Benign	Decreas e	8	DECREA SE	-1.4371507	No structural informati on	not affected (0.00)	Not affected (0.03)	not affected (0.00)	3.487	1
12	**D106 V**	**26.8**	**Benign**	**Decrea se**	**2**	**DECREA SE**	**- 0.5072736 3**	**No structura l informati on**	**not affected (0.00)**	**Not affected (-7.51)**	**Increase (182.93)**	**3.487**	**1**
13	D106 G	29.6	Benign	Decreas e	3	DECREA SE	-1.6391565	No structural informati on	not affected (0.00)	Not affected (-18.91)	Not affected (2.17)	3.487	1
14	V459 G	23.7	Benign	Decreas e	9	DECREA SE	-1.9494483	No structural informati on	not affected (0.00)	not affected (0.00)	not affected (0.00)	2.991	1
15	E491 K	31	Benign	Decreas e	8	DECREA SE	- 0.6299549 8	No structural informati on	not affected (0.00)	Not affected (-0.14)	Not affected (0.28)	4.348	1
16	E118 G	27.2	Benign	Decreas e	6	DECREA SE	-1.3814404	No structural informati on	not affected (0.00)	not affected (0.00)	not affected (0.00)	3.41	1
17	**D62Y**	**24.7**	**Benign**	**Decrea se**	**4**	**DECREA SE**	**-1.4666265**	**No structura l informati on**	**not affected (0.00)**	**Decreased (-76.79)**	**Not affected (32.39)**	**1.918**	**1**
18	D278 G	31	Benign	Decreas e	1	DECREA SE	-1.9486713	No structural informati on	not affected (0.00)	Not affected (-0.01)	not affected (0.00)	2.53	1
19	D612 Y	29.9	Benign	Decreas e	2	DECREA SE	- 0.9091356 1	No structural informati on	not affected (0.00)	Not affected (1.18)	Not affected (0.74)	4.102	1
20	**E578 K**	**29.9**	**Deleterio us**	**Decrea se**	**8**	**DECREA SE**	**-1.4887437**	**No structura l informati on**	**not affected (0.00)**	**Not affected (-0.14)**	**Not affected (0.23)**	**4.494**	**1**

Table S1: The results of second refinement step for identification of the most significant substitutions (for all 40 variants)

21	R45L	25.5	Benign	Decrease	8	DECREASE	-0.62002714	No structural information	not affected (0.00)	Not affected (0.28)	Not affected (-0.28)	2.815	1
22	K270N	24.9	Benign	Increase	1	DECREASE	-0.30434246	No structural information	not affected (0.00)	Not affected (0.13)	Not affected (-0.27)	2.815	1
23	R330C	32	Benign	Decrease	4	DECREASE	-0.97591216	No structural information	not affected (0.00)	Not affected (0.41)	Not affected (-0.25)	1.674	1
24	**R638L**	**29.2**	**Deleterious**	**Decrease**	**5**	**INCREASE**	**0.20503571**	**No structural information**	**not affected (0.00)**	**Not affected (2.88)**	**Not affected (-0.28)**	**4.102**	**1**
25	R638H	29.2	Benign	Decrease	8	DECREASE	-0.82572375	No structural information	not affected (0.00)	Not affected (0.18)	Not affected (-0.24)	4.102	1
26	R650P	28.9	Benign	Decrease	3	DECREASE	-1.4174138	No structural information	not affected (0.00)	Not affected (0.04)	Not affected (-0.28)	2.782	1
27	R650H	24.7	Benign	Decrease	8	DECREASE	-1.3029415	No structural information	not affected (0.00)	Not affected (2.54)	Not affected (-0.24)	2.782	1
28	**L648R**	**27.2**	**Deleterious**	**Decrease**	**8**	**DECREASE**	**-1.6859857**	**No structural information**	**not affected (0.00)**	**Not affected (-0.14)**	**Not affected (0.28)**	**3.559**	**1**
29	K55Q	25.2	Benign	Decrease	5	DECREASE	-0.34683895	No structural information	not affected (0.00)	Not affected (0.18)	Not affected (-0.27)	3.618	1
30	**R649C**	**32**	**Deleterious**	**Decrease**	**2**	**DECREASE**	**-0.71202154**	**No structural information**	**not affected (0.00)**	**Not affected (0.01)**	**Not affected (-0.28)**	**4.292**	**1**
31	**R638C**	**31**	**Deleterious**	**Decrease**	**2**	**DECREASE**	**-0.35557169**	**No structural information**	**not affected (0.00)**	**Not affected (0.14)**	**Not affected (-0.28)**	**2.442**	**1**

Table S1: The results of second refinement step for identification of the most significant substitutions (for all 40 variants)

32	**R638G**	**28.7**	**Deleterious**	**Decrease**	**6**	**DECREASE**	**-1.0760406**	**No structural information**	**not affected (0.00)**	**Not affected (0.14)**	**Not affected (-0.28)**	**2.442**	**1**
33	**R638S**	**27.2**	**Deleterious**	**Decrease**	**8**	**DECREASE**	**-0.65006129**	**No structural information**	**not affected (0.00)**	**Not affected (0.15)**	**Not affected (-0.28)**	**2.442**	**1**
34	**Q580P**	**26.3**	**Deleterious**	**Decrease**	**7**	**DECREASE**	**-0.81626228**	**No structural information**	**not affected (0.00)**	**Not affected (-1.24)**	**Not affected (-0.40)**	**3.731**	**1**
35	R100P	26.6	Benign	Decrease	4	DECREASE	-1.2420111	No structural information	not affected (0.00)	Not affected (0.14)	Not affected (-0.28)	2.608	1
36	N566K	24.5	Benign	Decrease	5	DECREASE	-1.2100999	No structural information	not affected (0.00)	Not affected (-0.20)	Not affected (2.15)	0.831	1
37	Q479H	25.3	Benign	Decrease	5	DECREASE	-0.89777933	No structural information	not affected (0.00)	Not affected (-0.00)	Not affected (0.03)	2.834	1
38	**R649H**	**26.7**	**Deleterious**	**Decrease**	**8**	**DECREASE**	**-1.1257388**	**No structural information**	**not affected (0.00)**	**Not affected (2.06)**	**Not affected (-0.24)**	**2.782**	**1**
39	**R105G**	**25.9**	**Benign**	**Decrease**	**8**	**DECREASE**	**-1.9637632**	**No structural information**	**not affected (0.00)**	**Increased (260.57)**	**Not affected (-0.28)**	**1.592**	**1**
40	R330L	26.6	Benign	Decrease	8	INCREASE	0.022820759	No structural information	not affected (0.00)	Not affected (0.18)	Not affected (-0.28)	3.621	0.996

Table S1: The results of second refinement step for identification of the most significant substitutions (for all 40 variants)
