# Supplementary material for: Prediction the functional impacts of highly deleterious non-synonymous variants of TSGA10 gene
Source: Mol Biol Res Commun. 2025;14(1):47–58. doi: 10.22099/mbrc.2024.49991.1977 (PMC11624612; doi:10.22099/mbrc.2024.49991.1977)
Supplement: Supplementary file 2 — Table S2 [file mbrc-14-47-s002.pdf]

Table S2: Confidence and coverage of the wild type and mutant models (Phyre2)

Amino acid change	Confidence (%)	Coverage (%)
**TSGA10**	99.7	93
**D62Y**	99.1	77
**R105G**	99.1	77
**D106V**	95.0	99
**D111Y**	99.1	77
**L524P**	99.6	94
**S563P**	99.3	76
**E578K**	99.6	94
**Q580P**	99.1	77
**R638L**	96.9	63
**R638C**	96.9	63
**R638S**	96.9	63
**R638G**	99.5	94
**L648R**	99.6	94
**R649C**	99.0	95.0
**R649H**	99.1	77
